# Investigator led trials: challenges and opportunities

**DOI:** 10.1186/1745-6215-12-S1-A94

**Published:** 2011-12-13

**Authors:** Peter Sandercock

**Affiliations:** 1 University of Edinburgh, Department of Clinical Neurosciences, Western General Hospital, Edinburgh EH4 2XU, UK

## 

Investigator led trials, whether of novel pharmaceutical agents, surgery or complex non-drug therapies, face a particular set of challenges. This is a selective review, based on personal observations and experiences of trials in my own field of stroke and clinical neuroscience over the past 32 years.

The guiding principles of clinical trials – all too often lost in the process of designing and managing clinical trials - are: trials should address a ‘burning question’; to provide sufficiently reliable evidence to answer that burning question (i.e. sufficient to change practice), trials need to be large; to achieve large sample sizes, trials need to be affordable and efficient in design and conduct; and, investigators need rigorously to apply the principles of the ‘business model’ of clinical trials to help overcome the many obstacles that inhibit trials.

From the 1980’s and into the early 2000’s, a series of very successful large-scale investigator- led trials in cardiovascular medicine and neuroscience had a substantial impact on clinical practice worldwide: the ISIS 1,2,3 & 4 trials in acute myocardial infarction, IST and CAST in acute stroke, CRASH in traumatic brain injury, ISAT in the management of ruptured intracranial aneurysms. All of these trials were conducted on remarkably modest budgets by today’s standards. After 2004, the regulatory environment changed significantly – especially for trials in the UK – with the implementation of the EU directive on clinical trials, the changes to UK ethical approvals system, and the introduction of the research governance framework. These changes have undoubtedly substantially raised the costs of, and delayed implementation of, clinical trials (and I would argue those costs and delays have disproportionately increased for investigator-led trials).

There is little disagreement that trials like those listed above simply could not be undertaken in the current regulatory environment. Clinical trials in the 21^st^ century must therefore adapt to the new environment (or die). However, for investigators planning their own trials now, there are grounds for a degree of optimism, since in the UK at least, several changes are underway that reduce the risk that investigator trials might become extinct. To name a few of these changes: the regulatory environment will ease a little; the number of registered clinical trials units in the UK has substantially increased to support new investigators, and the NIHR/MRC funding models are adapting to meet the needs of clinical trials; and, other changes are underway internationally that may also facilitate investigator-led trials.

